# Role of Heparin Irrigation in the Management of Superficial Burns with Special Reference to Pain Relief and Wound Healing: A Pilot Study

**DOI:** 10.7759/cureus.3157

**Published:** 2018-08-17

**Authors:** Chellappa Vijayakumar, Ravi Prabhu, M Senthil Velan, Vallinayagam Muthu Krishnan, Raja Kalaiarasi, Swetha T

**Affiliations:** 1 Surgery, Jawaharlal Institute of Postgraduate Medical Education and Research, Puducherry, IND; 2 General Surgery, Sri Lakshmi Narayana Institute of Medical Science, Puducherry, IND; 3 Ophthalmology, Sri Laksmi Narayana Institute of Medical Sciences, Puducherry, IND; 4 Otorhinolaryngology, Sri Lakshmi Narayana Institute of Medical Science, Puducherry, IND; 5 Obstetric and Gynaecology, Mahatma Gandhi Medical College and Research Institute, Pondicherry, IND

**Keywords:** burns, heparin, morbidity, quality of life, pain relief, wound irrigation, hospital stay, saline dressing, granulation tissue, necrotic tissue

## Abstract

Introduction

The objective of this study was to assess the effect of heparin irrigation in the management of superficial first and second degree burns with special reference to pain relief and wound healing.

Materials and methods

This pilot study was carried out over a period of 12 months in a tertiary care centre in South India. The study patients were divided into two groups: the heparin group and the saline control group. In the control group, the burn wound was irrigated with 100 mL of normal saline before the conventional dressing with silver sulfadiazine. In the heparin irrigation group, the wound was irrigated with heparin solution before the conventional dressing. Wound healing was assessed in terms of necrotic tissue score and granulation tissue score. Patient satisfaction in terms of patient satisfaction score, visual analogue scale (VAS) score, and length of hospitalization were compared between the two groups.

Results

A total of 40 patients were analysed in the study, 20 patients in each group. Both the groups were comparable with respect to age, gender, co-morbidities, body mass index (BMI), and degree of burns. Wound healing parameters like necrotic tissue score of six [40% vs. 50%; p = 0.024] and granulation tissue score of four [85% vs. 65%; p= 0.06] were significant in the heparin group compared to the control group. However, the difference was not statistically significant. The mean length of hospitalization between the two groups [10.5 days vs. 12.6 days; p = 0.74] were not statistically significant. Similarly, there was no statistically significant difference between the two groups with respect to the VAS pain score on the seventh dressing day [6.9 vs. 7.3; p= 0.321].

Conclusion

In comparison to saline irrigation, heparin irrigation would result in better wound healing in superficial first and second-degree burns. The length of hospital stay in days and VAS pain score on the seventh dressing day were not statistically significant between the two groups.

## Introduction

Treatments of first and second-degree burns are simple, comfortable for the patient and cheaper for the health care systems [1-3]. In developing countries like India, incidences of burns are on the rise. Superficial burns are more common than deep burns. They are extremely painful but carry substantially lower morbidity and mortality rates when compared to deep burns. Pain management and wound healing are the two crucial considerations while treating superficial burns. Recently, a wide range of topical anaesthetic drugs have been made available. Topical solutions which contain antiseptic, antibiotic, and growth factors properties are effectively used in superficial burns.

In India, sterile dressings are an expensive modality of treatment of burns. For this reason, patients deny admission at the hospital for a prolonged duration. Hence, it is necessary to use a single agent which provides adequate pain relief and accelerated wound healing. Heparin is a drug which satisfies both parameters. In addition to anticoagulation, heparin has anti-inflammatory, neo-angiogenic, and collagen restoring properties [4-6]. Several studies have reported that a large dose of heparin produces significant therapeutic results [5-6]. Very few centres have used topical heparin in the treatment of burns proving the efficacy of heparin in pain relief and wound healing. Few studies have reported heparin-related complications especially in the lungs and intestine [5-7].

Various studies have used heparin in deep burns [3,5]. In this study, the efficacy of heparin was tested in superficial burns on the grounds of wound healing and pain relief.

## Materials and methods

This pilot study was carried out over a period of one year in a tertiary care centre in South India. This trial included all patients admitted with the diagnosis of first and second-degree burns in the Department of Surgery. The study excluded patients with third-degree burns, liver disease, renal disorders, bleeding diathesis, allergy to heparin, active peptic ulcer, thrombocytopenia, and active bleeding or potential bleeding from trauma.

The Institutional Human Ethics Committee (IEC) approval was obtained for the study. The nature, methodology, and risks involved in the study were explained to the patient and informed consent was obtained. The information collected was kept confidential and the patient was given full freedom to withdraw at any point during the study. All provisions of the Declaration of Helsinki were followed in this study. Considering the feasibility and expected duration of the study, the sample size was calculated to be 30 in each group. The patients were divided into two groups: the heparin group (heparin irrigation) and the control group (saline irrigation).

**Figure 1:**
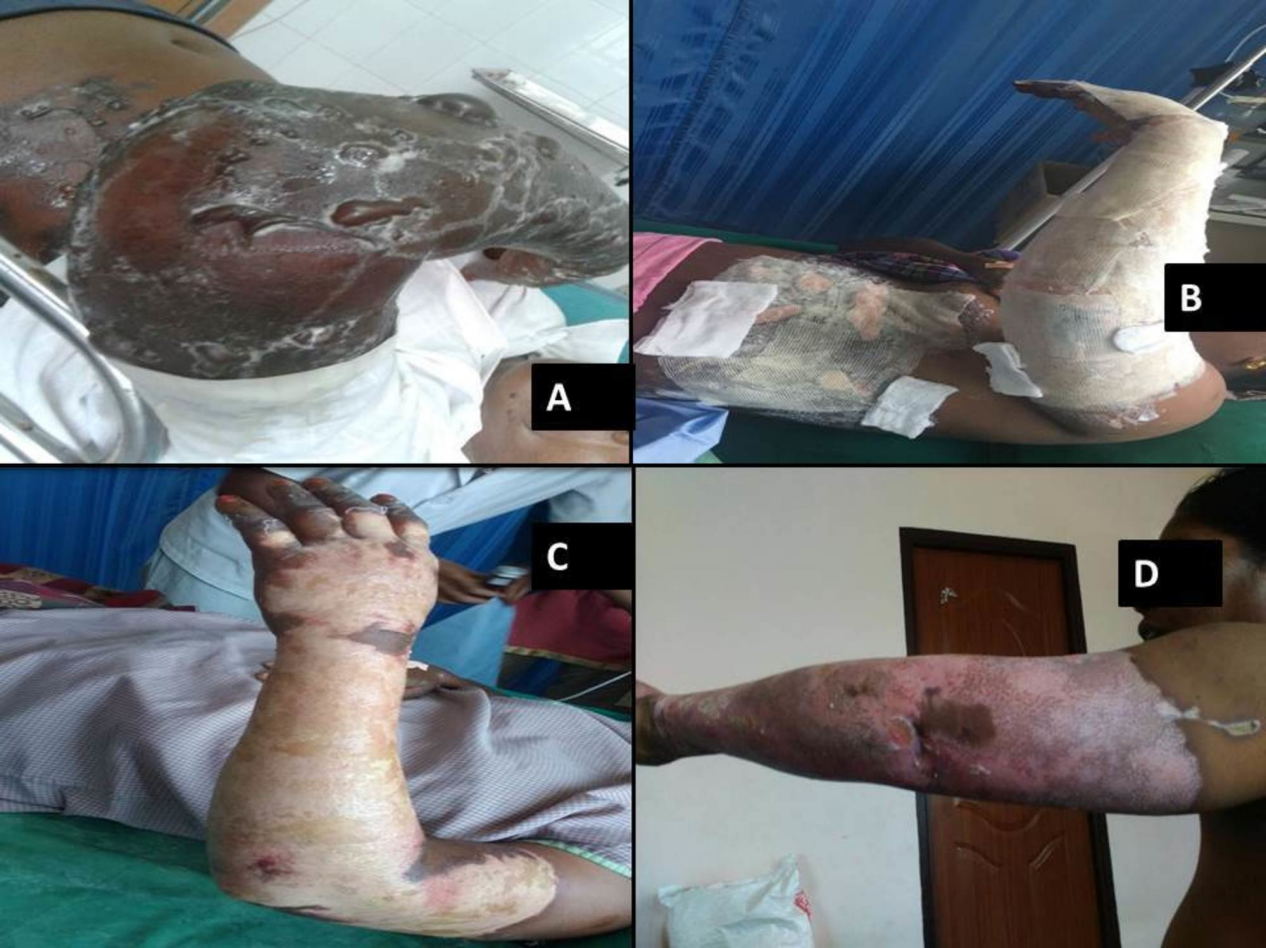
Heparin dressing in superficial burns A) superficial burns; B) heparin dressing; C) after one week of dressing; D) after four weeks of dressing.

The dose of heparin required for topical application was calculated to be 100,000 IU per 15% burn surface area (BSA) per day in 3 to 4 divided doses. The heparin was applied to the burnt surface drop by drop with a 20 mL syringe, until the pain was relieved and was repeated 2 to 4 times until blanching was noticed. On the second day, heparin was used twice a day, using a diminishing quantity over the next one week. The dressing was continued until complete healing was observed. The dressing was discontinued if the wound was considered healed (complete reepithelization) to the satisfaction of the treating surgeon (Figure 1).

The blisters were washed with heparin solution and not punctured. Additional blood investigations were done to test for bleeding time (BT), clotting time (CT), and activated partial thromboplastin time (APTT).

In the saline group, the wound was irrigated with 100 mL of normal saline before the conventional silver sulfadiazine dressing. In the heparin irrigation group, the wound was irrigated with heparin before the conventional dressing.

The wound was examined after 24 hours in both groups. Infected wounds were opened and packed. Wounds with impending infections were observed closely and opened in case of purulent drainage. The dressing was changed daily and the wound was examined in both groups.

The primary objectives were pain relief and healing of the burns wound. Relief of pain as recorded by a visual analogue scale (VAS) score was analysed between the groups. Wound healing parameters of necrotic tissue and granulation tissue score are as follows:

Percentage of wound covered with necrotic tissue using visual score

1. = 76%-100% wound covered with nonviable tissue

2. = 51%-75% wound covered with nonviable tissue

3. = 26%-50% wound covered with nonviable tissue

4. = 11%-25% wound covered with nonviable tissue

5. = 0%-10% wound covered with nonviable tissue

6. = No necrotic tissue

Percentage of wound covered with granulation tissue using visual score

1. = No granulation present

2. ≤ 25% of wound covered by granulation tissue

3. = 25%-74% of wound covered by granulation tissue

4. = 75%-100% of wound covered by granulation tissue

As given by a visual score, the percentage of necrotic tissue and granulation tissue covering the surface of the wound was analysed between the two groups at the beginning and the end of four weeks. Patient satisfaction towards wound healing, pain relief, and comfort of dressing were assessed by using the five-point Likert scale (excellent, good, fair, poor, and very poor) from the patient. These parameters were assessed and recorded in the specified proforma at the end of every week throughout the period of stay in the hospital. Length of hospitalization (in days) was also compared between the two groups.

Statistical analysis

Categorical variables like the degree of burns, gender, co-morbidity, body mass index (BMI), and percentage of burns were assessed by chi-square test. The variation in the degree of pain over time was analysed using Friedman’s test. For all statistical analysis, p value <0.05 was considered to be statistically significant.

## Results

**Figure 2:**
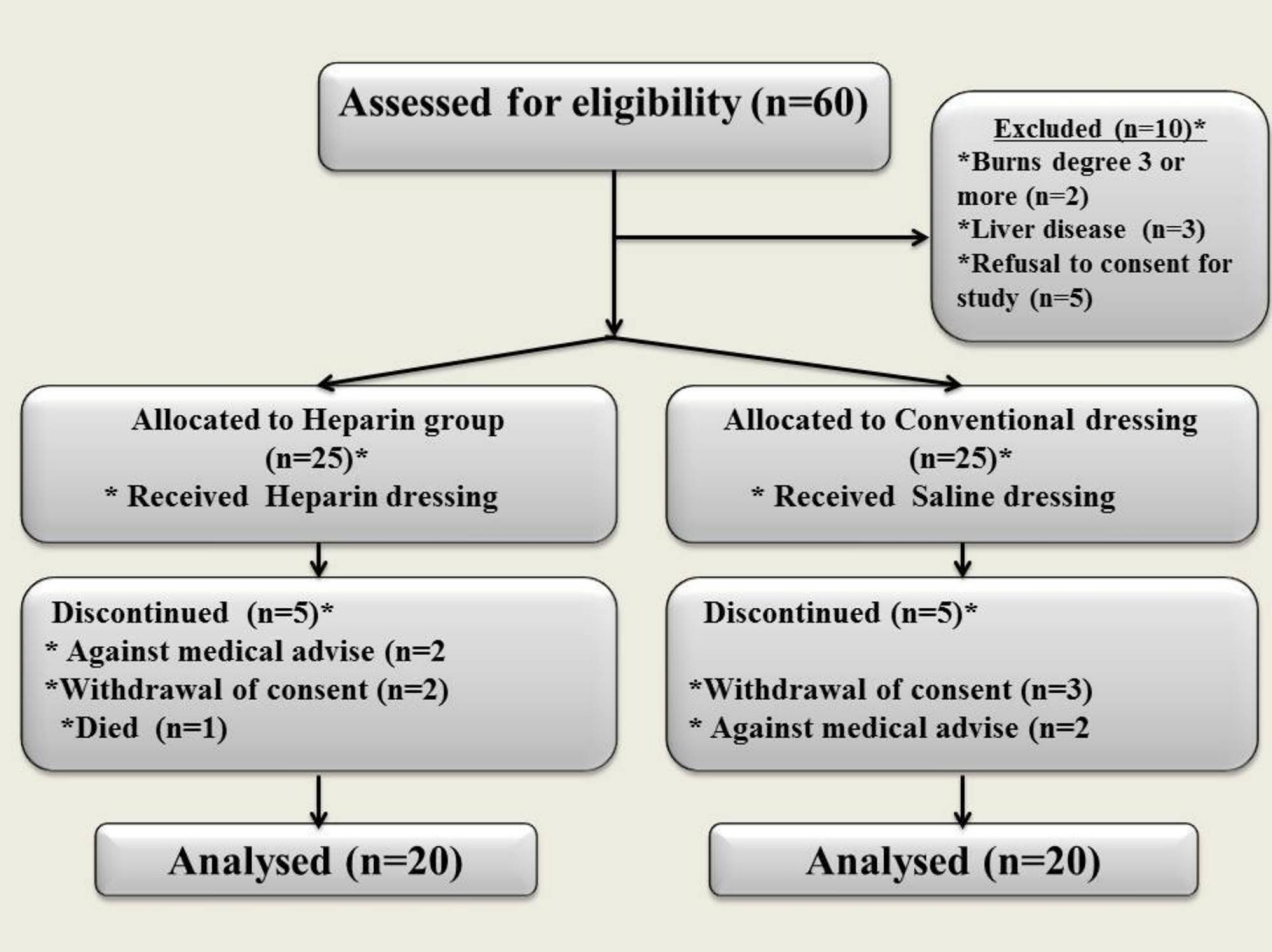
Study flow chart

A total of 60 patients were assessed for eligibility. Ten patients were excluded from the study. Ten patients were discontinued from the analysis. Nine patients withdrew themselves from the study and one patient succumbed to severe burns involving 72% BSA. A total of 40 patients were included in the study, 20 in the control group in which the wound was irrigated with normal saline followed by conventional silver sulfadiazine dressing and 20 patients in the heparin group in which wound was irrigated with heparin followed by conventional silver sulfadiazine dressing (Figure 2).

The age of patients in the control group varied from 27 to 65 with a mean age of 47.17±13.6. In the heparin group, it varied from 20 to 70 with a mean age of 51.34± 16.0. The comorbidities were distributed equally among the two groups. But the differences were not statistically significant (25% vs. 20%; p= 0.89). BMI between the study groups (26.54 vs. 25.34; p= 0.751) were comparable. But the difference was not statistically significant. Among the patients who were included in the study, 15 (75%) patients in the control group and 16 (90%) patients in the heparin group had first degree burns. The distribution of degree of burns were not statistically significant (p=0.33) between the groups (Table 1).

**Table 1:** Demographic parameters in the study groups

Demographic parameters	Heparin group (n= 20)	Control group (n= 20)	p-value
Age (Mean)	51.34	47.17	0.53
Gender- Male [N (%)]	13 (65 %)	12 (60%)	0.52
Co-morbidities [N (%)]	5 (25%)	4 (20%)	0.89
Body Mass Index (kg/m^2^)	26.54	25.34	0.751
Percentage of burns			
0 – 25% [N (%)]	16 (90%)	15 (75%)	0.33
25- 50% [N (%)]	2 (10%)	3 (15%)	
50 – 75% [N (%)]	2 (10%)	2 (10%)	

Degree of the burns

At the end of the fourth week, second-degree burns showed better healing in the heparin group compared to base line value (80% vs. 5%; p=0.023). This signified that most of the second-degree burns healed and transformed to first degree burns over four weeks. Similar conversion rate was also observed in the control group (90% vs. 40%; p=0.32). But this difference was not statistically significant (Table 2).

**Table 2:** Degree of the burns in the study groups

Degree of the burns N (%)	Heparin group (n=20)		Controls group (n=20)	
	Week 0	Week 4	Week 0	Week 4
Degree 1	04 (20%)	19 (95%)	02 (10%)	12 (60%)
Degree 2	16 (80%)	01 (5%)	18 (90%)	08 (40%)

Necrotic tissue score

A necrotic tissue score of six means no necrotic tissues were present in the wound. At the end of the fourth week, it (50% vs. 40%; p=0.024) was observed in both groups. The difference was statistically significant (Table 3).

**Table 3:** Percentage of necrotic tissue over the burns between the study groups * No ulcers were found on that particular week.

Necrotic tissue score N (%)	Heparin group (n=20)		Controls group (n=20)	
	Week 0	Week 4	Week 0	Week 4
1	2 (10%)	0*	0*	0*
2	11 (55%)	0*	6 (30%)	1 (5%)
3	5 (25%)	2 (10%)	14 (70%)	0*
4	2 (10%)	2 (10%)	0*	3 (15%)
5	0*	6 (30%)	0*	8 (40%)
6	0*	10 (50%)	0*	8 (40%)

Granulation tissue score

A granulation tissue score of four means 75%-100% of the wound is covered by granulation tissue. At the end of the fourth week, it (85% vs. 65%; p= 0.06) was observed in both groups. The difference was again statistically significant (Table 4).

**Table 4:** Percentage of granulation tissue over the burns between the study groups * No ulcers were found on that particular week.

Granulation tissue score N (%)	Heparin group (n=20)		Controls group (n=20)	
	Week 0	Week 4	Week 0	Week 4
1	9(45%)	0*	7(35%)	0*
2	11(55%)	1(5%)	12 (60%)	1(5%)
3	0*	2(10%)	1(5%)	6(30%)
4	0*	17(85%)	0*	13(65%)

VAS score for pain

**Figure 3:**
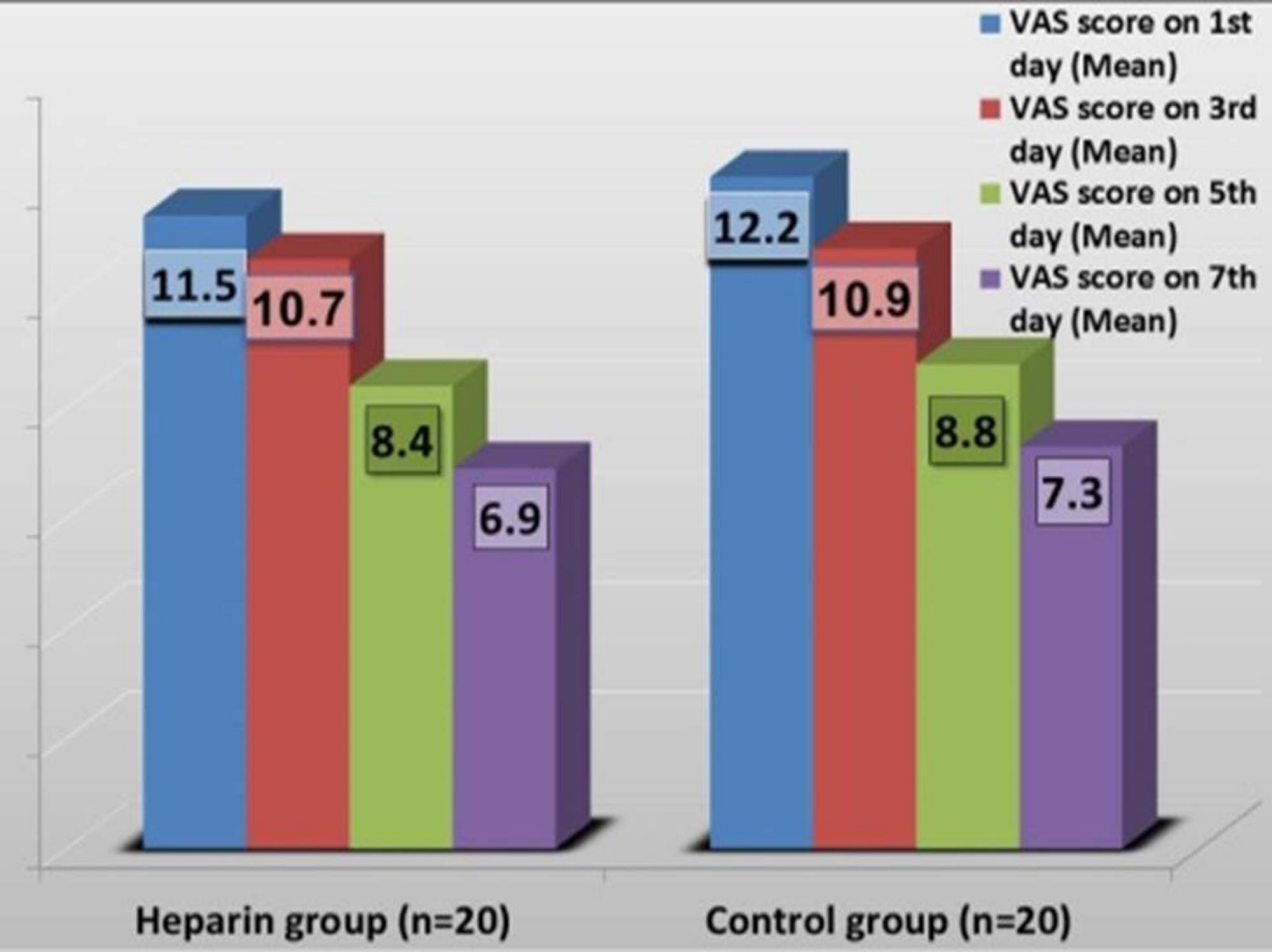
Visual analogue scale (VAS) score between the study groups

Mean VAS score on the seventh day between the two groups were not significant (6.9 vs. 7.3; p=0.321). When compared to base line value, the mean VAS score on the seventh day was significantly reduced in the heparin group (11.5 vs. 6.9; p= 0.032). Similar results were also observed in the control group (12.2 vs. 7.3; p= 0.573). But this difference was not statistically significant (Figure 3).

Patient satisfaction score in dressing

**Figure 4:**
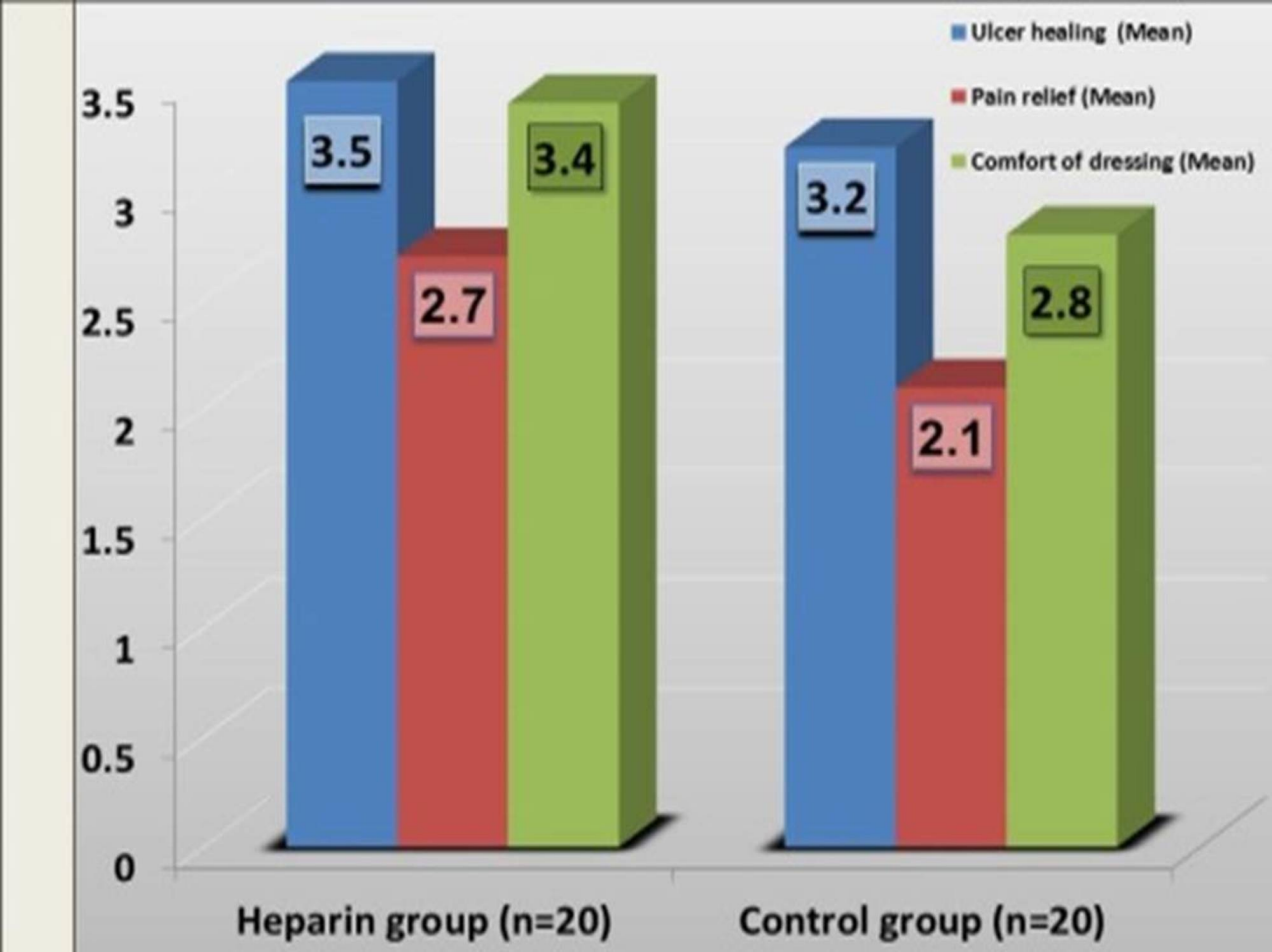
Patient satisfaction score between the study groups

Patient satisfaction score in wound healing (3.5 vs. 3.2), pain relief (2.7 vs. 2.1), and comfort of dressing (3.4 vs. 2.8) were significant but this difference (p˃0.05) was not statistically significant (Figure 4).

Length of hospital stay (days)

**Figure 5:**
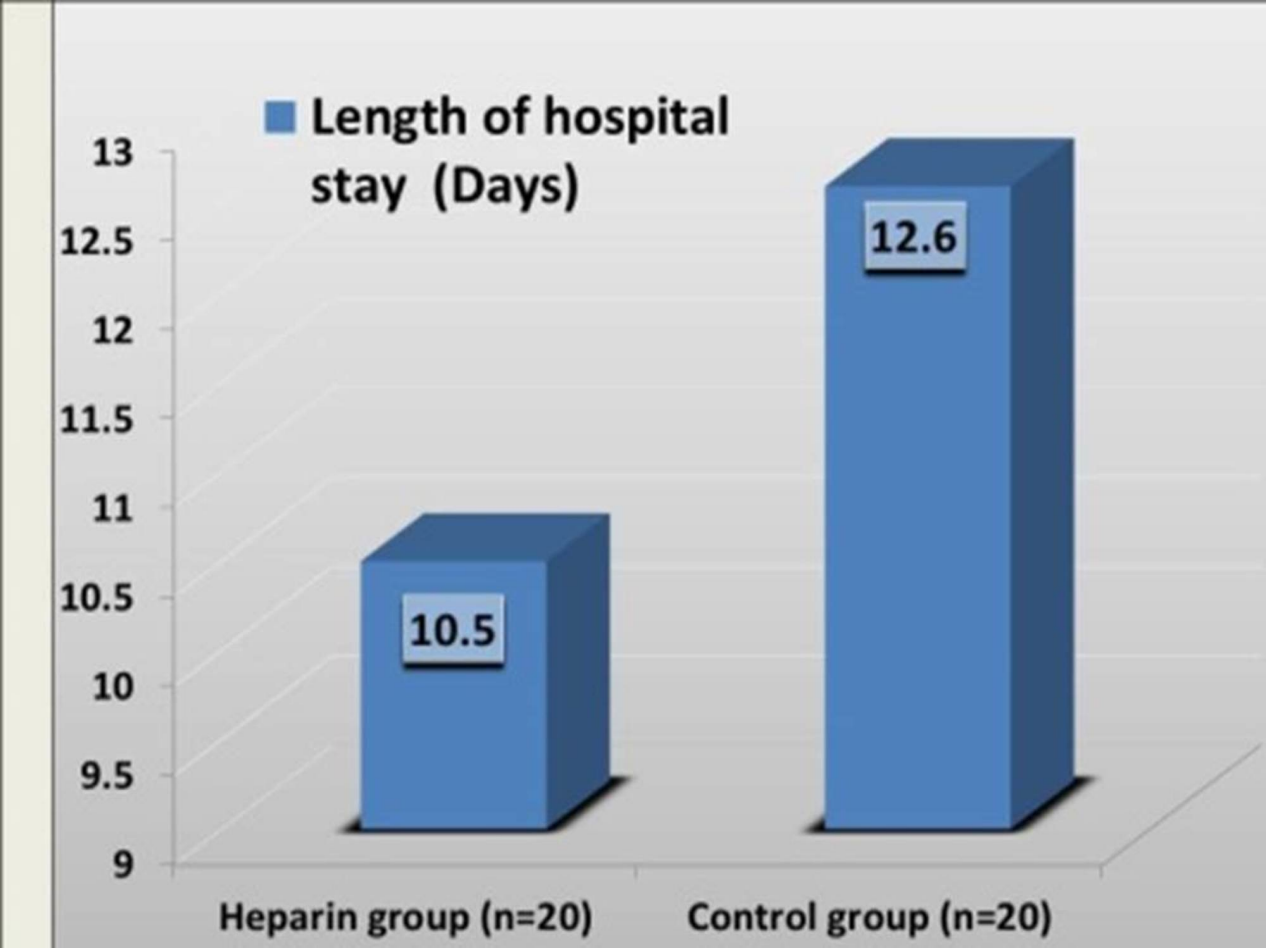
Length of hospital stay (days) in study groups

Length of hospital stay between the study groups (10.5 days vs. 12.6 days; p= 0.74) was not statistically significant (Figure 5).

## Discussion

Heparin is effective in the management of burns [2]. Heparin acts by the inhibition of blood clotting, improvement in blood circulation, decrease inflammation, increase in granulation, and reduction of scarring [4]. Superficial burns are usually accidental. Due to its anti-inflammatory properties, heparin produces a dramatic reduction in pain, inflammatory edema, and redness. Pain is assessed by VAS, largely based on the patient’s perspective. The amount of heparin used is directly proportional to the size and degree of burns. Pain is the most important parameter which restricts the patient’s mobility and prolongs the duration of stay in the hospital.

According to various studies, prolonged inflammation and stagnation of neutrophils is characteristic of burn wounds. Secretory products of neutrophils, such as elastase, cathepsin G, and proteinases, are harmful to wound healing because they damage the extracellular matrix and growth factors and further recruit neutrophils to the burns area. Heparin inhibits the action of these secretory elements by its electrostatic action [5-8].

In this study, demographic parameters like age, sex, comorbidities, BMI, and percentage of burns were comparable in both groups. Saliba MJ et al. demonstrated similar findings in his study [9-10].

Degree of burns

After four weeks of heparin dressing, only 5% (n=1) were observed to be second-degree burns and the rest 95% (n=19) had healed. This difference (60%; n=12) was statistically significant in comparison with the control group. Due to its neoangiogenesis property, heparin prevents extension of burns and results in a remarkable reduction in the degree of burns [5-7].

Necrotic tissue score

Necrotic tissue is the main parameter of wound healing which was assessed by a visual score. The presence of necrotic tissue is the evidence of ongoing sepsis. At the end of the fourth week, the burn wounds of eight patients (40%) had necrotic tissue. Prior studies have suggested that topical heparin has the propensity to increase blood flow mediated by an increase in neoangiogenesis of the ischemic tissue [8-10]. This provides direct evidence wound healing by topical heparin.

Granulation tissue score

Presence of granulation tissue indicates progressive healing of burn wounds. In this study, a granulation tissue score of six was present in 17 patients (85%) in the heparin group. A reduction in bacterial load in superficial burns may be another explanation for the increased granulation tissue seen in this study [11]. Presence of adequate granulation tissue enables earlier discharge from the hospital. This partly explains why antibiotics may not be required in superficial burns unless it is grossly infected.

VAS score for pain

Most of the patients in the study complained of severe pain. Conventional treatment of burns with silver sulfadiazine takes a longer time to achieve pain reduction and healing (re-epithelisation). Topical anaesthetic drugs are needed to decrease the pain which necessitates removal of the adhered dressing every day, disturbing the healing environment. The heparin study group reported less pain while consuming less analgesic medication, as compared to the patients treated with the conventional dressing group. A number of studies reported in the literature have proved the beneficial effect of heparin in terms of pain relief [12-14].

Patient satisfaction score in dressing

Patient satisfaction remains the most important goal of any novel treatment modality. Patients provided their opinion about pain relief, ulcer healing, and comfort of dressing which was translated to a score. Nine patients withdrew their consent at the beginning of the study. This usually happens when a new intervention is tested. Hence, we analysed patient perspective parameters separately at the end of the study. Overall patient satisfaction was higher in the heparin group compared to the saline group. To the best of our knowledge, this is the first study to analyse the patient satisfaction parameters in the management of burns using heparin.

Length of hospital stays (days)

Length of hospital stay in days was remarkably lesser in the heparin group. Skin grafting was done in two patients (10%) in both the groups. This was the reason for the delay in discharge from the hospital. A relatively cost-free treatment is the other reason for prolonged stay especially in patients with poor family support. Similar results were observed in many studies [11,13].

Burns are disasters which not only affect the quality of life but also subject the patient to emotional stress. The development of newer treatment resources could possibly modify this scenario. In this study, a new intervention using topical heparin was used to relieve pain and enhance healing.

Limitations

Since the study was a pilot study, only a limited number of patients were included. Larger sample size with longer follow up could possibly substantiate the wound healing and sepsis-related complication. Heparin is expensive which may be a limiting factor due to poor affordability in a private tertiary care centre. Topical heparin (in the form of a spray) has properties of simplicity, comfort and reasonable cost. Its use in burn management in high volume burn centres may be fruitful. This study did not analyse scar cosmesis and sepsis-related parameters. Further studies are needed to substantiate the therapeutic use of heparin in the treatment of superficial burns [14].

## Conclusions

To conclude, even as research for newer modalities in the treatment of superficial burns continues, conventional dressing modalities still appear to provide a good clinical outcome. Although numerous studies support the use of topical heparin in the management of burns, many are yet to define the appropriate treatment and outcome.

## References

[1] Saliba MJ (2001). Heparin in the treatment of burns: a review. Burns.

[2] Robson MC, Kucan JO, Paik KI, Heggers JP (1979). The effect of heparin on dermal ischaemia after burning. Burns.

[3] Masoud M, Wani AH, Darzi MA (2014). Topical heparin versus conventional treatment in acute burns: a comparative study. Indian J Burns.

[4] Azizkhlan RG, Azizkhkan JC, Zetter BR, Folkman J (1980). Mast cell heparin stimulates migration of capillary endothelial cells in vitro. J Exp Med.

[5] Costa NF, Verçoza AJ (2007). Treatment of burns with heparin. Burns.

[6] Carr J (1979). The anti-inflammatory action of heparin: heparin as an antagonist to histamine, bradykinin and prostaglandin E1. Thromb Res.

[7] Agbenorku P, Fugar S, Akpaloo J, Hoyte-Williams PE, Alhassan Z, Agyei F (2013). Management of severe burn injuries with topical heparin: the first evidence-based study in Ghana. Int J Burns Trauma.

[8] Ferreira Chacon JM, Mello de Andrea ML, Blanes L, Ferreira LM (2010). Effects of topical application of 10,000 IU heparin on patients with perineal dermatitis and second-degree burns treated in a public pediatric hospital. J Tissue Viability.

[9] Saliba MJ, Dempsey WC, Kruggel JL (1973). Large burns in humans, treatment with heparin. JAMA.

[10] Zayas GJ, Bonilla AM, Saliba MJ (2007). Heparin reduced mortality and sepsis in severely burned children. Ann Burns Fire Disasters.

[11] Church D, Elsayed S, Reid O, Winston B, Lindsay R (2006). Burn wound infections. Clin Microbiol Rev.

[12] Lu J, Xu T, Yang M, Xu XW, Wu B (2011). Heparin for the treatment of burns. Cochrane Database Syst Rev.

[13] Griffith GC, Bogss RP (1964). The clinical usage of heparin. Am J Cardiol.

[14] Oh SJ, Jang YC, Lee JW (2007). Clinical use of heparin in acute second degree burns. Burns.

